# Technology Matters: Mental health apps – separating the wheat from the chaff

**DOI:** 10.1111/camh.12363

**Published:** 2019-12-13

**Authors:** Aislinn Bergin, E. Bethan Davies

**Affiliations:** ^1^ NIHR MindTech MedTech Co‐operative Institute of Mental Health School of Medicine University of Nottingham Nottingham UK

Young people are more connected to their phones than ever—by 12 over 80% of young people in the UK have their own smartphone. Whilst this rate of access may concern some, it does provide a clear opportunity for young people to access mental health support, advice and tools in a timely and engaging way in the form of apps. ‘Mental Health App’ is a catch‐all term that is used to describe applications (‘apps’) for mobile devices with a variety of purposes and functions.

For young people, this can include (but is not limited to) mood symptom assessment, monitoring and tracking, strategies to aid mood or symptoms like anxiety, and educating users about mental health. As the number and complexity of these apps increase, so does their potential relevance to practitioners. There are now apps that provide interventions directly to the user (such as BlueIce (Stallard, Porter, & Grist, 2018), which was developed by clinicians in partnership with young people with experience of self‐harm) and apps that aim to support and enhance the therapeutic process (such as Power Up (Edbrooke‐Childs et al., 2019), which aims to help children better communicate their needs to services).

With many thousands of apps claiming to address mental health and well‐being and more apps coming on the market every month, how can practitioners identify those that may be beneficial? Simply searching app stores using keywords will result in large numbers of apps, many of which are likely to be at best irrelevant and at worst dangerous or examples of modern‐day quackery. So how can practitioners decide which they can confidently recommend to young people?

## How to judge health apps

When considering a mental health app, there are three main aspects that practitioners should consider. First and most important are the clinical components: Is the information, advice or intervention accurate and in line with current evidence or good practice, and is it safe for young people to use? Secondly, technical aspects must also be considered and whether the app has clear and adequate processes for data security. Finally, its usability; Will young people be able to use it, and will it work properly on the devices (and data plans) that young people have access to?

### Judging clinical

As noted above, mental health apps vary considerably in terms of their functionality and intended benefits and this has been recognised by regulatory bodies such as the FDA (U.S. Food and Drug Administration) and MHRA (Medicines and Healthcare products Regulatory Agency) who now classify apps into different classes or tiers which determine the level of assessment a particular product requires.

Much like the self‐help books that practitioners can recommend, mental health apps that provide information, advice and strategies do not require any formal assessment. However, apps that make claims of diagnosis or treatment or are expensive or complex are required to validate their claims and register as a medical device with the appropriate regulatory body. We are currently not aware of any mental health apps for children and young people that have been registered as a medical device.

Most practitioners find out about potentially relevant apps through word of mouth and are likely to rely more on the opinions and recommendations of a colleague or endorsement of a professional body, rather than looking for clinical evidence of its effectiveness. This is due in part to the small number of app evaluations that have been conducted but also explains why many app developers focus more on improving the ratings and reviews of their apps as it is likely to be significantly quicker, easier, cheaper and less‐risky than conducting a formal evaluation.

Many apps described as ‘evidence‐based’ utilise principles of evidence‐based therapy and would perhaps be better described as ‘theory‐based’. However, whilst theory‐based can mean that clinical expertise is embedded into the app’s development, it may not fully account for differences in delivery via a new technology.

### Judging technical

The data collected by mental health apps are often personal and sensitive, and this is a particularly important issue for apps aimed at children and young people. There are clearly data protection issues that must be considered, but this is one of the more difficult areas to assess. Whilst we might hope to rely on the claims about data security made by apps, the majority of top‐rated depression apps were recently found to share data with a third party, with many not disclosing this information in their privacy policy (Huckvale, Torous, & Larsen, 2019).

### Judging usability

Whilst the clinical aspects of apps are important, it is also essential to consider usability. This means ensuring that the design and content of the app are appropriate. For instance, cartoons might not be the best option for older teenagers and, because apps can be accessed from anywhere, the emergency contacts may be based in another country. Whilst mental health apps such as BlueIce ensure usability through close collaboration with young people, this will not always guarantee engagement. Reviews on app stores can be a useful place to find information about what people do or do not like about an app, but it is also critical to discuss these with young people themselves. They can provide more in‐depth insights, like what devices they use and whether they can access the Internet easily as well as what they enjoy and feel motivated to use. Additional factors to consider include the associated costs (both up front and ongoing), the device and data requirements as well as its suitability for specific types of young people (Fleming et al., 2019). But with all these issues to consider and with many thousands of mental health apps available, it is clearly not realistic for practitioners or organisations to assess each app individually.

## Curated app libraries: finding a needle in a haystack

In an attempt to solve this issue, there have been numerous attempts to develop health app libraries, which provide a curated list of apps that have been judged to meet certain standards. These have had varying success. For instance, the first iteration of the UK’s NHS apps library was suspended in 2015 following publication of research that raised concerns about data security (Huckvale, Prieto, Tilney, Benghozi, & Car, 2015) and the evidence base (Leigh & Flatt, 2015) of the apps listed. There are now several consumer‐facing app libraries, many with a formal review process, including ORCHA, OurMobileHealth and a new beta version of the NHS apps library. However, these cover all areas of health and so do not include information on the latest research evidence for mental health apps or provide practitioners with guidance on how these specific apps should be used to improve services and care.

The American Psychiatric Association has developed an evaluation model for mental health apps that considers safety and privacy, evidence, ease of use and interoperability (Torous et al., 2018). It is welcome that professional bodies are starting to provide guidance and advice which is specific to mental health, and in the future, we may see organisations such as the APA or the BPS or ACAMH develop an approach similar to peer review where they publish lists of apps relevant to their members. Until then, practitioners and organisations need to use existing curated libraries and their own judgement to ensure that any mental health apps they recommend have been appropriately assessed for clinical and technical quality, usability and relevance.

## Implications for practitioners

This piece is intended to be a call to action for practitioners. Mental health apps are here to stay and have the potential to be important tools if practitioners and young people understand them and know how to choose and use them.

It is important for practitioners to recognise that young people will be using apps and will find them in all kinds of ways based on a variety of recommendations. There are limits, therefore, to what practitioners can be responsible for in terms of telling young people what they should or should not be using. Instead we recommend talking to young people about apps and letting them guide you as much as you do them, learn what they like to use, explore it professionally, and blend it into the conversations about their care.

We finish with some recommendations for those considering mental health apps. We suggest looking at the apps listed on curated app libraries and those used by colleagues and consider which might be useful for your own practice. Use app assessments like those produced by the American Psychiatric Association to help you make judgements and critically analyse what is available, much as you would peer review research. Consider the evidence that is already available through app evaluations and systematic reviews, which are increasingly considering usability as well as clinical effectiveness. And finally, get involved and talk about apps with colleagues, patients and families to find out what they are using and what it is about those apps that are particularly helpful to them.

## Ethical approval

No ethical approval was required for this article.
